# Effect of *Scenedesmus* sp. CHK0059 on Strawberry Microbiota Community

**DOI:** 10.4014/jmb.2205.05016

**Published:** 2022-06-30

**Authors:** Gyeongjun Cho, Gyeong Seo Jo, Yejin Lee, Youn-Sig Kwak

**Affiliations:** 1 Research Institute of Life Sciences, Gyeongsang National University, Jinju 52828, Republic of Korea; 2 Division of Applied Life Science (BK21 Plus), Gyeongsang National University, Jinju 52828, Republic of Korea; 3 Department of Plant Medicine, Gyeongsang National University, Jinju 52828, Republic of Korea

**Keywords:** Biostimulator, *Chlorella fusca*, microalgae, *Streptomyces*

## Abstract

Microalgae are photosynthetic cyanobacteria and eukaryotic microorganisms, mainly living in the water. In agriculture, numerous studies have been conducted to utilize microalgae as a biostimulant resource. *Scenedesmus* has been known to be one such microalga that can promote plant growth by secretion of auxin or cytokinin hormone analogs. However, no research has been performed on the effect of microalgae treatment on plant microbiota communities. This study was conducted to investigate the mode of action of microalgae as biostimulants in a plant microbiota perspective by using *Scenedesmus* sp. CHK0059 (also known as species *Chlorella fusca*), which has been well documented as a biostimulant for strawberries. The strawberry cultivar Keumsil was bred with Seolhyang and Maehyang as the parent cultivars. Using these three cultivars, microbiota communities were evaluated for changes in structural composition according to the CHK0059 treatment. CHK0059-treated Seolhyang, and CHK0059-untreated Maehyang were similar in microbial diversity in the endosphere. From a microbiota community perspective, the diversity change showed that CHK0059 was affected by the characteristics of the host. Conversely, when CHK0059 treatment was applied, populations of *Streptomyces* and *Actinospica* were observed in the crown endosphere.

## Introduction

As the global population is expected to increase to 9.8 billion by 2050, food shortages caused by environmental destruction and gaps in agricultural lands are expected to become more and more severe [1]. For these reasons, many researchers have been studying sustainable agriculture. Microalgae have potential value as a bioresource [2] for solving food problems because they can produce biomass through photosynthesis, cause less concern about environmental pollution, and reproduce easily in a relatively short period of time. Among various microalgae species, the genera *Chlorella*, *Chlamydomonas*, and *Scenedesmus* are under active study for use in sustainable agriculture [3, 4]. *Chlorella*, *Scenedesmus*, and *Chlamydomonas* are all known to produce diverse bioactive metabolites and have a high potential to be beneficial for various crops [5-7].

*Scenedesmus* sp. CHK0059, the strain used in this study, is a single cell known by the species name *Chlorella fusca*, the genus of which was reclassified based on physiological, biochemical, and genetic characteristics [6]. Genus *Scenedesmus* is also an evolutionarily important microalga because some of the mitochondrial codons are non-standard, placing it in a different class Chlorophyceae [9]. Extracts of *Scenedesmus obiliquus* have been evaluated to increase the seed germination, root development, and cotyledon expansion of certain plants [10]. As a plant biostimulant, CHK0059 is well documented for its ability to enhance strawberry growth and marketability [11]. Induced systemic resistance (ISR) response of cucumber to *Colletotrichum orbiculare* was reported [12], and D-lactic acid produced by CHK0059 has been found to induce ISR in *Arabidopsis* [12]. Additionally, in *Erinus alpines*, CHK0059 exhibited an anti-aging effect [13].

In general, multicellular organisms have precise interactions between the organisms and their surrounding or inner microorganisms. The term ‘microbiota’ comes from seeing one organic and complex relationship as an integrated complex organism [14]. Bacteria, archaea, fungi, protists, and viruses are members of plant microbiota and are evolutionarily selected based on long-standing interactions between the host and other related members [14]. With the advancement of next-generation sequencing technology, the plant microbiota community structure and composition process are being revealed [15]. Plant microbiota are reported to affect the plant life cycle by producing hormones, metabolites, nutrients, tolerance against abiotic stress, systemic acquired resistance, and induced systemic resistance against various pathogens [16].

The strain CHK0059 has been shown to have significant positive effects on many crops, and strawberries (*Fragariae × ananassa*) are also reported to be affected by CHK0059 treatment [11]. However, the expression of stress tolerance-related genes in plants by microalgae treatment has not occurred. Therefore, the plant biostimulant effect of microalgae is likely to be implemented through changes in the microbiota community or an enhanced population of keystone microbes for plant health. This study focuses on the changes or responses of the plant microbiota community to better understand the mode of action of the plant biostimulant CHK0059, a known, sustainable agricultural material, In addition, we tried to find changes that occur in common or each host cultivar by evaluating variations according to the host cultivar.

## Materials and Methods

### Strawberry Cultivation and Application of CHK0059

Strawberry plant cultivars Maehyang, Seolhyang, and Keumsil were cultivated in a greenhouse at Gyeongsang National University, Jinju, Republic of Korea (35°9'17" N 128°5'56"E). Seolhyang is the most widely cultivated strawberry cultivar in Korea, and Maehyang is mainly cultivated in the southern region of Korea for export. Keumsil was developed for export by complementing the shortcomings of Maehyang as a crossbreed of Seolhyang and Maehyang. Therefore, this study attempted to understand microbial community structure resulting from microalgae treatment of three strawberry cultivars. CHK0059 strain was obtained from the Rural Development Administration (Korea), and the microalgae culture medium is a product from F&B Nature Co., Ltd., (Korea). The CHK0059 was initially inoculated at 1 × 10^6^ cells/ml in the media and cultivated at 28°C for 6 days. The light condition was 120 μmol/m^2^/s for 24 h a day, and air circulation was 2 L/min. To calculate the concentration after the CHK0059 harvest, cell numbers were counted using a hemocytometer (CELLOP, Korea). CHK0059 at an average 1.82 × 10^7^ cells/ml concentration was diluted 9:1 with distilled water and treated 100 ml/plant twice a week, and the same volume of 1× Hogland solution was treated once a week. The strawberry plants were cultivated for 40 days, and they were drenched with CHK0059 10 times during the cultivation period. After 40 days, strawberry rhizosphere soil, root tissue, and crown tissue samples were collected to analyze microbiota community structures (*n* = 5).

### DNA Extraction and PCR for Pyrosequencing

CHK0059-treated and -untreated strawberry rhizosphere, root endosphere, and crown endosphere were used for microbiota community analyses. Before the isolation of microbiota DNA, the strawberry rhizosphere was collected by shaking after tapping and removing the excess soil that was not close to the roots. The root and crown were then dissected. Next, the root was washed with flowing water, and the crown was cut by about two to five millimeters. To prevent contamination due to this series of tasks, the tissues were disinfected in 70% ethyl alcohol for 30 s, and then 1% NaOCl for 30 s. The disinfected tissues were dried on a clean bench for 1 h followed by rinsing with autoclaved distilled water. DNA extraction was carried out by FastDNA SPIN Kit for Soil (MP Biomedicals, USA) with 0.5 g rhizosphere or 0.4 g endosphere of each tissue. Extracted DNA quality and quantity were checked at 230 nm, 260 nm, and 280 nm light wave using a spectrometer (NanoDrop 2000C, Thermo Scientific, USA), and the DNA concentration was set to 5-8 ng/μl. Amplification of 16S rRNA V4 region using PCR was proceeded by Illumina adapter linked primer, 515F (5’-TCGTCGGCAGCGTCAGATGTGTATAAGAGACAGG TGCCAGCMGCCGCGGTAA) and 805R (5’-GTCTCGTGGGCTCGGAGATGTGTATAAGAGACAGGAC TACHVGGGTATCTAATCC), the final dose of which was 0.4 μM. To restrain amplification of the mitochondrial and chloroplast sequences of the host, 0.75 μM of plastid peptide nucleic acid (5’-GGCTCAACCCTGGACAG) and mitochondrial peptide nucleic acid (5’-GGCAAGTGTTCTTCGGA) clamps were used. The DNA polymerase and its buffer solution were KAPA HiFi HotStart ReadyMix (Roche, Switzerland). Temperature conditions of the chain reactions were 95°C for 3 min in initial denaturation, 95°C for 30 s in repeated denaturation, 55°C for 30 s in repeated annealing, 72°C for 30 s in repeated elongation, and 72°C for 5 min in final elongation. The repeated chain stage was 25°C in the rhizosphere sample, and 28-30°C in the endosphere sample. The amplified V4 region was provided by Macrogen Inc. (Republic of Korea) and sequenced by Illumina MiSeq 2 × 300 bp.

### Amplicon Sequence Variant (ASV) Cluster and Strawberry Microbiota Community Analyses

The fastq files of the MiSeq 2 × 300 bp results were handled and computed by R (4.1.3 version) on Ubuntu20.04 in the windows subsystem for Linux 2 on Windows 11 installed on a personal computer with 32 GiB DDR4 RAM and AMD Ryzen 9 5950X. The reads were conducted by denoising amplicon sequence variants and clustered using DADA2 (1.20.0 version) [17] and the rarefaction curve is presented (Fig. S1). Each ASV was identified by IDTAXA classifier [18] in DECIPHER (2.20.0 version) with SILVA 138 SSU. The community was analyzed with R program. Alpha diversity and beta diversity were calculated by stats (4.1.3 version), vegan (2.6-2 version), and phyloseq (1.36.0 version) in R packages. The different abundances of bacteria in the CHK0059-treated and untreated groups were reckoned by ANCOM-BC (analysis of compositions of microbiomes with bias correction) developed by Lin and Peddada [19]. All visualized graphs are based on ggplot2 (3.3.5 version).

## Results

### Commonly Dominant Bacteria at the Family Level on Three Cultivars of Strawberry

**Fig. 1:**
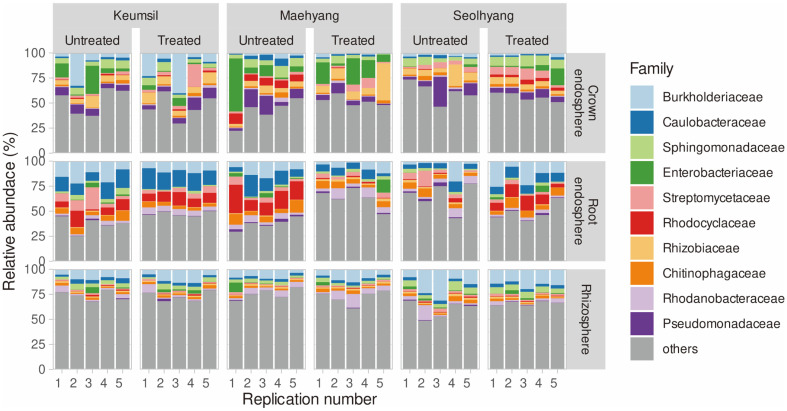
Relative abundance. The colors display the dominant top 10 families. The panel is split according to strawberry cultivar, CHK0059 treatment, and the host composition.

Structural shifting of the microbiota communities of the strawberry rhizosphere, root endosphere, and crown endosphere was observed to determine the effect of CHK0059 treatment on each strawberry cultivar. The major species of bacteria showed no significant difference between CHK0059-treated and -untreated groups regardless of strawberry cultivar. The top 10 bacteria families were *Burkholderiaceae*, *Caulobacteraceae*, *Sphingomonadaceae*, *Enterobacteriaceae*, *Streptomycetaceae*, *Rhodocyclaceae*, *Rhizobiaceae*, *Chitinophagaceae*, *Rhodanobacteraceae*, and *Pseudomonadaceae* (Fig. 1). The abundance of the top 10 families was 50.6% in Keumsil crown endosphere, 57.8% in Keumsil root endosphere, 26.8% in Keumsil rhizosphere, 53.2% in Maehyang crown endosphere, 49.9%in Maehyang root endosphere, 26.3% in Maehyang rhizosphere, Seolhyang crown endosphere, 41.5% in Seolhyang root endosphere, and 43.2% in Seolhyang rhizosphere (Fig. 1).

### CHK0059 Effect on Diversity in Strawberry Bacterial Community

**Fig. 2:**
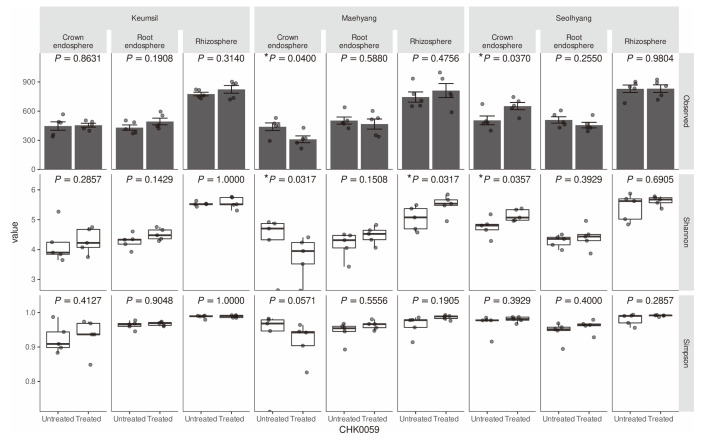
Alpha diversity of crown endosphere, root endosphere, and rhizosphere each cultivar. Chao1 has normality and variances homogeneity, but Shannon and Simpson have no normality. Therefore, the observed Student’s *t*-test compares ASV richness and Shannon and Simpson indices are compared by Wilcoxon signed-rank test between CHK0059-treated and untreated. The signed-rank comparison test excludes outliers based on IQR methods. The standard error is indicated by the bar in the Chao1 panel. (*p < 0.05)

**Fig. 3:**
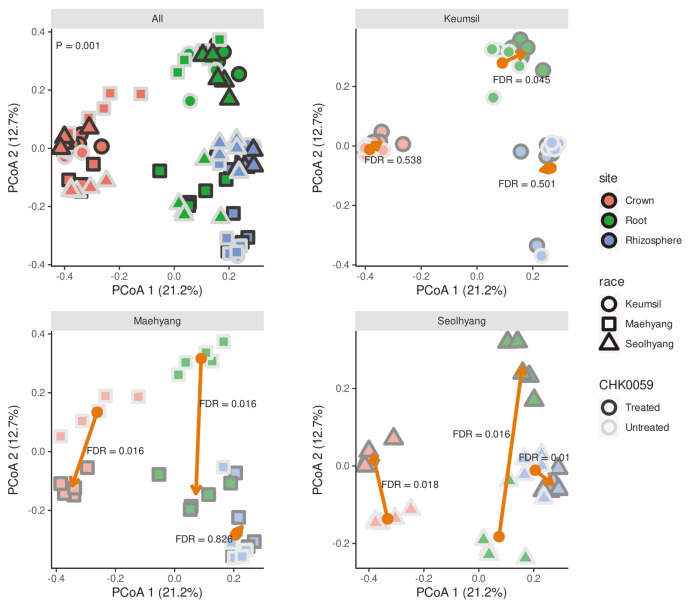
Principal coordinates analysis of beta diversity using Bray-Curtis distance at ASV levels. Colors indicate where it was sampled. Cultivars are distinguished by point shape. The point border darkness displays CHK0059 treatment or not. The Keumsil, Maehayng, and Seolhyang panel orange arrow lines are visualized by treatment average to untreated average for each composition on the coordinate plane. The FDR values besides the arrows are calculated by pair-wise permutational multivariate analysis of variance. The adjusted *p*-value method is a false discovery rate. The crown and root endosphere arrows in Maehyang and Seolhyang have opposite directions between Maehyang and Seolhyang. The percentage label of the axis is a ratio of each eigenvector centrality of the axis to the sum of eigenvector centrality including all axes not shown.

The effect of CHK0059 on microbiota diversity was investigated. As alpha diversity (richness and evenness of each sample) is observed, ASV means mainly richness, while Shannon’s and Simpson’s diversity indices pertain to both richness and evenness appropriately. The crown endospheres of cultivars Maehyang and Seolhyang differed significantly in the observed ASV and the Shannon diversity index. However, the indices showed that Maehyang was increased and Syeolhyang was decreased by the CHK0059 treatment (Fig. 2). By the Shannon index, the Maehyang rhizosphere under the CHK0059 treatment was significantly higher than in untreated samples. The alpha diversity effect of CHK0059 was not observed in Keumsil (Fig. 2). Observation of beta diversity (diversity compared between two groups) was progressed by Bray-Curtis distance at each ASV. The distances among samples were displayed to dimensional compression by PCoA (Fig. 3). The beta diversity distribution was different in at least one group based on the cultivar and CHK0059-treated or -untreated group. A post hoc, pair-wise ERMANOVA was performed. The results show that the distribution of Keumsil root, Maehyang root, Maehyang crown, and Seolhyang’s composition differed between the CHK0059-treated and –untreated groups. Moreover, the positions of the CHK0059-treated group and the CHK0059-untreated group on the PCoA2 axis were in opposition (Fig. 3). The crown and root endophytic bacterial communities showed a more significant response than the rhizosphere community by CHK0059 treatment. These results showed that CHK0059 affected the diversity of the strawberry bacterial communities, and the effect was different for each cultivar and their respective compositions.

**Fig. 4:**
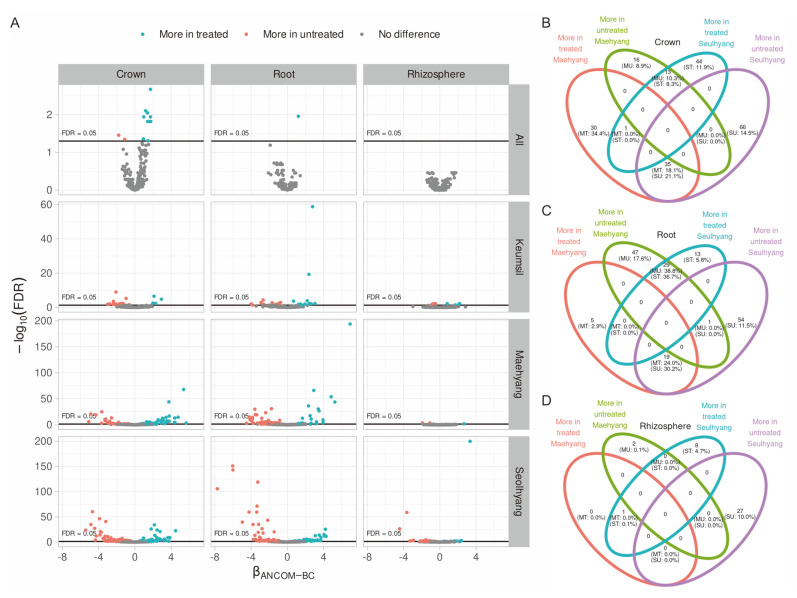
ANCOM-BC at taxonomic group level. (**A**) Y-axis is significant, and X-axis is a natural log-linear model value of abundance. Blue and redpoint mean that a taxonomic group is more in treated and untreated samples (FDR < 0.05). Difference is less in the rhizosphere than other sites and is more in Maehyang and Seolhyang than Keumsil. Summaries ANCOM-BC using Venn diagram and tile plot. The number in a circle without a bracket means the count of the taxonomic group in the crown endosphere (**B**), root endosphere (**C**), and rhizosphere (**D**). Percentage in the bracket under the taxonomy group counter, which is described as points in Maehyang and Seolhyang panels in the ANCOM-BC volcano plot, indicates the average relative abundance of the groups (MT: Maehyang treated, MU: Maehyang untreated, ST: Seolhyang treated, SU: Seolhyang untreated).

To comprehend the difference of the CHK0059 impact on bacterial community diversity of each cultivar, the abundance of the taxonomic group, which was computed by the most detailed identification result of each ASV, was compared by ANCOM-BC (analysis of compositions of microbiomes with bias correction) (Fig. 4A). Significantly, taxonomy groups on Keumsil that differed according to the CHK0059-treated or -untreated groups had the smallest number among the three cultivars. On the other hand, Keumsil's parent cultivars, Maehyang and Seolhyang, had more different taxonomy groups. To better understand the response of the microbiota community to CHK0059 from the ANCOM-BC result, the difference between parents was organized by Venn diagrams (Figs. 4B-4D). In the rhizosphere, overwrapped taxonomy was hardly observed but the crown and root endosphere had taxonomy mainly overwrapped by untreated Maehyang treated Seolhyang, and treated Maehyang untreated Seolhyang (Fig. 4D). On average, the relative abundance of the intersection groups was 10.3% in untreated Maehyang crown, 8.3% in treated Seolhyang crown, 18.1% in treated Maehyang crown, and 21.1% in untreated Seolhyang crown (Fig. 4B). Also, 38.8% in untreated Maehyang root, 36.7% in treated Seolhyang root, 24.0% in treated Maehyang root, and 30.2% in untreated Seolhyang root (Fig. 4C). Taxonomy groups of more among CHK0059-treated or -untreated groups were almost nil. This indicated that the CHK0059 effect differed among the cultivars. However, no change in strawberry phenotype by CHK0059 treatment was observed in this experiment.

### Increase or Decrease of ASV by CHK0059 Treatment

To discover the increases of bacteria in the CHK0059-treated or –untreated groups among the three strawberry cultivars, ANCOM-BC was also conducted for all cultivars (Fig. 4A). Treatment with CHK0059 had no significant effect on the rhizosphere. However, *Brevibacterium* was more abundant in the treated groups, mostly in the root endosphere, and *Candidatus*, *Koribacter*, *Actinospica*, *Brevibacterium*, *Streptomyces*, *Nubsella*, WD2101 soil group, *Rhodopila*, *Limnobacter*, *Luteibacter*, and *Stenotrophomonas* were more abundant in the treated groups, commonly in the crown endosphere. *Pirellula* and *Hyphomicrobiaceae* were more abundant in the untreated group in the crown. In addition, the Pearson correlation analysis with the corresponding taxa confirmed that *Streptomyces* and *Actinospica* had a positive correlation with the CHK0059 treatment because the changes were more commonly observed in the crown (Table 1, Fig. S2).

## Discussion

Microalgae are well reported as effective plant biostimulants and biological control agents against fungal pathogens, nematodes, and even viruses [6, 7]. This study was designed to evaluate the effectiveness of plant microbiota community altering by *Scenedesmus* CHK0059, which has been well documented as a plant biostimulant [11], although its mode of action has not been defined. Despite that microalgae are used in numerous ways in agriculture, very limited studies have been conducted on the changes or responses in the microbiota community caused by microalgae treatment.

Metagenomic 16S rRNA amplicon analyses revealed that the dominant bacterial taxa in the tissue composition of strawberry were *Burkholderiaceae*, *Caulobacteraceae*, *Sphingomonadaceae*, *Enterobacteriaceae*, *Streptomycetaceae*, *Rhodocyclaceae*, *Rhizobiaceae*, *Chitinophagaceae*, *Rhodanobacteraceae*, and *Pseudomonadaceae*, regardless of whether CHK0059 treatment was applied or not. The effect of CHK0059 on alpha diversity was observed to have changed the richness of the crown endosphere microbiota cultivars Maehyang and Seolhyang. When evaluated from the perspective of beta diversity, distribution in PCoA results showed division by composition. Intriguingly, changes in microbiota community structure by application of CHK0059 were detected in the crown endosphere, but not in the rhizosphere, regardless of the strawberry cultivars. The results support a previous finding that the application *Chlorella vulgaris* did not affect bacterial diversity in the bean rhizosphere [20]. Unfortunately, no analysis of the endosphere microbiota community of beans was attempted due to the influence of microalgae in the study [20].

Based on the result of PERMANOVA, the 18 groups based on the cultivars, compositions, and CHK0059 treatment were judged that at least one of them was distinguished at the Bray-Curtis distance. In pair-wise PERMANOVA as a post hoc test of the results, treated and untreated endosphere sample distribution of each cultivar Seolhyang and Maehyang, the parent cultivars of Keumsil, was distinguished significantly, and their PCoA results showed that their positions were opposite. On the other hand, interestingly, CHK0059-untreated Seolhyang and CHK0059-treated Maehyang, CHK0059-treated Seolhyang, and CHK0059-untreated Maehyang were similar in endosphere composition. For a more complete picture, the three cultivars were analyzed by differential analysis at the taxa level and Venn diagrams and tile plots summarized the results. These results presented the microbiota community change as more apparent; Keumsil was less affected by CHK0059 than the other two cultivars. Seolhyang and Maehyang had many abundances of different taxonomies. Diversity and Venn diagram results clearly showed that the endospheres of cultivars Seolhyang and Maehyang had the opposite trend with and without the CHK0059 treatment. The different reaction phenomena may be due to genetic differences between the cultivars.

Microalgae are known to promote plant growth and protect plants from biotic and abiotic stresses by producing plant hormones, D-lactic acid, toxin, antibiotic metabolites, and ISR [7]. However, these mechanisms alone are not sufficient to explain that microalgae have a wide range of beneficial functions in various plants. Therefore, to ascertain CHK0059 as a plant biostimulant from the standpoint of interaction between CHK0059 and the plant microbiota community, microbes that increase or decrease in common were searched on the basis of CHK0059 application. *Brevibacterium* was detected as an abundant bacterium in the root endosphere by CHK0059 treatment. Application of *Brevibacterium diminature* with microalgae mixture enhanced rice yield up to 1.2-fold compared to no application [21]. In the crown endosphere, ten and two taxonomy groups were more abundant in the CHK0059-treated samples and more abundant in the untreated samples, respectively. The correlations among the cultivars and the CHK0059 application were analyzed by Pearson correlation based on a centered log ratio for deeper comprehension. Only two specific bacteria, *Streptomyces* and *Actinospica*, were correlated with the CHK0059 treatment. In many cases, the correlation analysis does not specify causality well enough, but this study was planned with the CHK0059 treatment as the independent variable, and the dependent variable was community changes. Therefore, it was judged that the increase of *Actinospica* and *Streptomyces* was due to the CHK0059 treatment. Although these results indicated that CHK0059 has an effect of increasing the population density of certain microbes in the community, more in-depth research is required to address which mechanisms may involve the buildup of the beneficial microbe density in the system.

This study displayed two major microbiota community changes in strawberry by CHK0059 treatment. One was the bacteria varied according to the cultivar during the CHK0059 treatment or not, which was probably a case in which the bacteria phase changed due to the interaction between the CHK0059 and the plant. Also, the beta diversity interactions of CHK0059, which showed opposite tendency between Seolhyang and Maehyang, and the beta diversity interaction of Keumsil, which fell somewhere in the middle, suggest the possibility that the beta diversity was considered as a kind of phenotype. The other was the common increase in bacterial taxa by the CHK0059 treatment. CHK0059 probably interacted directly without the host factor, and CHK0059 was helping *Streptomyces* and *Actinospica* to prevail in the bacterial community. In addition, *Actinospica* and *Streptomyces*, which belong in Actinomycetia, were reported to be important for tolerance and phytoremediation of heavy metal pollution [22]. *Streptomyces* also reported well on plant growth promotion [23-27] because it was classified among the top 10 families. In conclusion, we present a new model theory in which microalgae contribute to plant growth by increasing the density of certain beneficial microbes among plant microbiota community members.

## Figures and Tables

**Table 1:** Pearson’s correlation after centered log-ratio transformation with abundance of commonly more and less existing taxonomy groups.

Compared group1	Compared group2	ρ	*P* value	Significant
*Actinospica*	*Streptomyces*	0.231	0.236	
*Actinospica*	WD2101 soil group	-0.014	0.949	
*Actinospica*	CHK0059	0.471	0.011	*
*Streptomyces*	WD2101 soil group	0.445	0.023	*
*Streptomyces*	CHK0059	0.447	0.013	*
WD2101 soil group	CHK0059	0.287	0.155	

If the abundance is zero, it is difficult to judge whether it does not exist or is rare. For this reason, the sample of zero abundance is excluded. If the sample size with that taxonomy group did not exceed 25, it was excluded from the analysis because of β error. (*p < 0.05)
