# Erratum to: Effect of *Scenedesmus* sp. CHK0059 on Strawberry Microbiota Community

**DOI:** 10.4014/jmb.2022.3211.1496

**Published:** 2022-11-28

**Authors:** Gyeongjun Cho, Gyeong Seo Jo, Yejin Lee, Youn-Sig Kwak

**Affiliations:** 1 Research Institute of Life Sciences, Gyeongsang National University, Jinju 52828, Republic of Korea; 2 Division of Applied Life Science (BK21 Plus), Gyeongsang National University, Jinju 52828, Republic of Korea; 3 Department of Plant Medicine, Gyeongsang National University, Jinju 52828, Republic of Korea

In the article titled Effect of *Scenedesmus* sp. CHK0059 on Strawberry Microbiota Community, the funding acknowledgment was partially incorrect as published.

## Original version

Acknowledgments

This work was supported by the Rural Development Administration (PJ014934).

## Corrected version

**Acknowledgments**

This work was supported by the Rural Development Administration (PJ015641).

